# Oxygen uptake and heart rate responses to 4 weeks of RPE-guided handcycle training

**DOI:** 10.1007/s00421-023-05210-7

**Published:** 2023-04-29

**Authors:** Michael J. Hutchinson, Thomas A. W. Paulson, Christof A. Leicht, Hunter Bennett, Roger Eston, Victoria L. Goosey-Tolfrey

**Affiliations:** 1 British Paralympic Association, London, UK; 2 Exercise and Health Sciences, Loughborough University, Loughborough, UK; 3 UK athletics, Birmingham, UK; 4 Allied Health and Human Performance, University of South Australia, Adelaide, Australia; 5 Alliance for Research in Exercise, Nutrition and Activity (ARENA), University of South Australia, Adelaide, Australia

**Keywords:** Self-regulated, Upper body, Exercise, Intensity, Reliability

## Abstract

**Purpose:**

To investigate the efficacy of using Ratings of Perceived Exertion (RPE) to prescribe and regulate a 4-week handcycle training intervention.

**Methods:**

Thirty active adults, untrained in upper body endurance exercise, were divided into three groups to complete a 4-week intervention: (i) RPE-guided training (n = 10; 2 female), (ii) power output (PO)-guided (n = 10; 2 female) training, or (iii) non-training control (n = 10; 4 female). Training groups performed three sessions of handcycling each week. Oxygen uptake (${\dot{\text{V}}}O_{2}$), heart rate (HR), and Feeling Scale (FS) rating were collected during training sessions. RPE-guided training was performed at RPE 13. PO-guided training was matched for percentage of peak PO per session, based upon that achieved by the RPE-guided training group.

**Results:**

There were no differences in percentage of peak $\dot{V}O_{2}$ (66 ± 13% vs 61 ± 9%, p = 0.22), peak HR (75 ± 8% vs 71 ± 6%, p = 0.11) or FS rating (1.2 ± 1.9 vs 0.8 ± 1.6, p = 0.48) between RPE- and PO-guided training, respectively. The average coefficient of variation in percentage of peak HR between consecutive training sessions was 2.8% during RPE-guided training, and 3.4% during PO-guided training.

**Conclusion:**

Moderate-vigorous intensity handcycling exercise can be prescribed effectively using RPE across a chronic training intervention, suggesting utility for practitioners in a variety of rehabilitation settings.

## Introduction

The American College of Sports Medicine recommends that adults engage in a minimum of 150 min week^−1^ of moderate intensity exercise, 75 min week^−1^ vigorous intensity exercise, or a combination of the two (ACSM 2020). Though recommendations are based on achieving a combination of exercise duration and intensity, exercise intensity can be difficult to monitor. Direct measures of intensity include percentage of maximal oxygen uptake (%$\dot{V}O_{2\max }$) and maximal heart rate (%HR_max_), with moderate and vigorous intensity classified as 46–63% $\dot{V}O_{2\max }$ or 64–76% HR_max_, and 64–90% $\dot{V}O_{2\max }$ or 77–95% HR_max_, respectively (ACSM 2020). But given the need for specialist equipment to measure intensity directly, alternatives are required to facilitate exercise prescription away from a controlled, laboratory environment.

One method of guiding training intensity is using Ratings of Perceived Exertion (RPE). RPE-guided training allows individuals to set their workload based on how hard they perceive the exercise, providing an equipment free method of prescribing exercise intensity in a variety of settings (Bok et al. 2022; Borg and Noble 1974). Additionally, as exercising at a self-selected intensity has been shown to elicit a more positive affective response than prescribed exercise of the same intensity (Rose and Parfitt 2007; Hamlyn-Williams et al. 2014), RPE-guided exercise may also improve exercise adherence (Williams et al. 2008).

RPE-guided training anchored at RPE 13 (somewhat hard) (Borg 1998) has been shown to increase $\dot{V}O_{2\max }$ during an 8-week running intervention in sedentary adults (Parfitt et al. 2012). The training intensity was also shown to satisfy the description of “moderate” intensity (61–64% $\dot{V}O_{2\max }$) (Parfitt et al. 2012). Similarly, a 4-week cycling intervention, with training anchored at RPE 13, led to an average response of 64% HR reserve during training (Ilarraza et al. 2004). While these findings support RPE-guided training, they are currently lacking thorough analysis of the physiological responses to RPE-guided training sessions, and how those responses change over the duration of a training intervention. It remains unknown if prolonged RPE-guided training leads to consistent $\dot{V}O_{2}$ and HR responses equivalent to “moderate” or “vigorous” exercise.

Additionally, the application of RPE-guided training in handcycling exercise requires further attention. Given the feasibility associated with RPE-guided exercise, it may offer a viable tool in a variety of exercise settings, including the rehabilitation and training of individuals with lower limb impairments (e.g., amputees and spinal cord injuries). Such situations necessitate the use of upper-limb dominant exercises, such as handcycling, to improve cardiovascular fitness. To date, RPE has been demonstrated as an accurate way of prescribing handcycling exercise intensity in comparison to individualised power outputs equating to between 40 and 70% of $\dot{V}O_{2\max }$ in able-bodied individuals (Paulson et al. 2013a) and those with spinal cord injury (Paulson et al. 2013b; Goosey-Tolfrey et al. 2010). However, this previous research has only been conducted in acute exercise settings, and has not been explored across a longitudinal RPE-guided training intervention.

As such, the primary aim of this study was to investigate the physiological and perceptual responses to a 4-week RPE-guided handcycle exercise training intervention in able-bodied participants. A secondary aim was to compare the affective response to RPE-guided and power output (PO)-guided training. It was hypothesised that training at RPE 13 would produce a physiological response that would satisfy the description of “moderate” to “vigorous” intensity exercise across a 4-week training intervention (ACSM 2020). Furthermore, it was hypothesised that RPE-guided training would lead to a more positive affective response than PO-guided training.

## Methods

### Experimental design

Thirty physically active participants, untrained in upper body endurance exercise, volunteered to take part in the study and provided written informed consent. Participants were screened using a standardised university health screening questionnaire based upon the Physical Activity Readiness Questionnaire (PAR-Q) (Thomas et al. 1992) to ensure they had no co-morbidities that could be exacerbated by maximal exercise (i.e., cardiovascular disease and hypertension), or any existing musculoskeletal injuries to the upper body that could impact upon their ability to complete the intervention. The study was approved by the Loughborough University (human participants) ethics sub-committee (ethics protocol number R15-P067) and conducted in accordance with the Declaration of Helsinki. Participants completed a 4-week handcycle training intervention with a single exercise testing session pre and post. A 4-week intervention was considered long enough to observe consistent physiological and perceptual responses to handcycling, based upon previous research in cycling (Ilarraza et al. 2004). All trials were performed using the same handcycle (Invacare Top End Force 3, Elyria, OH, USA), that was attached to a Cyclus 2 ergometer (Avantronic Richter, Leipzig, Germany). During the first session the handcycle was configured to the participant’s comfort while allowing slight elbow flexion at the furthest point in the crank cycle. This configuration was recorded and applied for all subsequent sessions. Prior to the initial exercise testing session participants recorded their 24-h dietary intake and replicated this before the post-training maximal tests. Participants were instructed to avoid strenuous exercise, alcohol, and caffeine, for 24 and 6 h preceding maximal trials. During all exercise trials and sessions handcycling cadence was kept constant on an individual basis. The participant’s preferred cadence was established in the warm-up of their first trial, where they were invited to experiment with various cadences and asked to choose the one that felt most comfortable. This cadence was then maintained during test and training sessions.

### Training intervention

Participants were randomly allocated to the RPE-guided training (n = 10, 8 male, 2 female; 24 ± 4 years; 78.3 ± 18.7 kg; 1.77 ± 0.10 m) or control (CON; n = 10, 6 male, 4 female; 21 ± 4 years; 67.3 ± 5 kg; 1.73 ± 0.11 m) groups. The PO-guided (n = 10, 8 male, 2 female; 25 ± 3 years; 74.2 ± 12.3 kg; 1.77 ± 0.10 m) training group was recruited after the completion of the RPE-guided and CON groups, as a convenience sample ensuring similarity between existing groups. CON were asked to maintain their normal exercise regime for the four week period, while all participants in the RPE- and PO-guided groups continued their habitual exercise regime and also performed 30 min of supervised handcycle exercise, three times per week, for four weeks. Subsequently, training sessions are referred to by the week and session number (1.1, 1.2, 1.3, 2.1 etc.) with the first number denoting the week of the intervention, and the second number denoting the session number within that week. Participants in the RPE-guided group performed all training at an overall RPE (RPE_O_) equal to 13, or “somewhat hard,” on Borg’s 6–20 RPE scale (Borg 1998), which has previously shown efficacy at prescribing exercise in a variety of populations and settings (Bok et al. 2022). RPE_O_ encompassed the degree of physical strain felt in the exercising musculature and cardiorespiratory systems (Marcora 2010). Participants selected their work rate and were instructed to change the PO as often as needed to maintain the required RPE, whilst maintaining their preferred cadence. The PO was changed up or down by 5 W at a time by using buttons attached to one of the cranks, with 5 W being the smallest change that could be made on the ergometer. Participants were blinded to PO during exercise to minimise the likelihood of exercise intensity being influenced by anything other than RPE.

The PO-guided training group performed each session at the %PO_peak_ obtained from the pre-training graded exercise test (GXT), equivalent to the group-average produced by the RPE-guided group. The average %PO_peak_ was calculated for each individual session for the RPE-guided group, and then used to prescribe the intensity of exercise for the PO-guided training group on a session-by-session basis. As such, the RPE-guided training group was completed before the PO-guided group started training.

For both training groups, HR (RS400, Polar, Kempele, Finland) and PO were monitored continuously each training session. $\dot{V}O_{2}$ (Metalyzer 3B, Cortex, Leipzig, Germany). Blood lactate concentration ([BLa] (Biosen C-line monitor (EKF diagnostics, Barleben, Germany) was monitored during the very first session of the intervention (1.1), and in the final session of each week (1.3, 2.3, 3.3, 4.3). For [BLa] a 20 µl capillary blood sample was taken from the ear lobe. Gas exchange variables were monitored continuously and [BLa] was measured every 10 min. Perceptual measures of peripheral RPE (RPE_P_), central RPE (RPE_C_), and RPE_O_, using Borg’s 6–20 RPE Scale (Borg 1998), as well as Feeling Scale (FS) rating (Hardy and Rejeski 1989), were recorded at 5 min intervals throughout each training session. The RPE_P_ and RPE_C_ were defined as the degree of physical strain felt in the exercising musculature and cardiorespiratory systems, respectively (Marcora 2010). All perceptual measures were obtained upon instruction by the primary investigator, whereby participants verbally reported their RPE whilst looking at the scale. Prior to each trial participants were read standardised verbal instructions on the use of the relevant RPE scale (Borg 1998) and were specifically asked to focus on the degree of how hard, heavy, and strenuous the physical task was (Marcora 2010). For RPE_P_ participants were asked to focus on the RPE of the exercising muscles, on their heart and lungs for RPE_C_, and for a combination of them all for RPE_O_. Regarding FS, participants were instructed to consider how good or bad they felt at that present moment and provide a rating, based upon standardised verbal instructions (Parfitt et al. 2012). All participants were familiarised with the scales prior to commencing their initial exercise testing session.

Excluding the first 4 min of exercise (until a steady state was reached), all variables were averaged across each session and calculated as percentages of peak from the pre-training GXT. During training sessions for both groups, all data other than the elapsed time, cadence and perceptual scales were blinded from participants.

### Peak exercise testing

Participants completed a maximal GXT pre and post the training intervention using the same handcycle (Invacare Top End Force 3, Elyria, OH, USA) attached to a Cyclus 2 ergometer (Avantronic Richter, Leipzig, Germany) used during the training intervention. The two GXT were performed at the same time of day (± 1 h) (Hill et al. 1989). Prior to the GXT, participants completed a 5-muinute warmup at a self-selected speed. To maintain a test duration of approximately 10 min, starting PO ranged from 20 to 40 W depending on the participants self-reported baseline fitness levels, and was increased by 10 W·min^−1^ until participants reached volitional exhaustion or could not maintain within 20 rpm of their preferred cadence across a 1-min stage despite verbal encouragement to do so. $\dot{V}O_{2}$, HR and PO were collected throughout tests, then subjected to 30 s rolling averages, with the greatest single value taken as the peak response (Robergs et al. 2010).

### Statistical analyses

Analysis was performed using IBM SPSS Statistics 23 (SPSS Inc., Chicago, IL.). Data are presented as mean ± SD, and statistical significance was accepted at p < 0.05. Data were checked for normal distribution using the Shapiro Wilk statistic.

The $\dot{V}O_{2}$, HR and PO for each training session were calculated as a percentage of peak from the pre-training GXT, and with the perceptual responses, were averaged across each session. To assess the %$\dot{V}O_{{2{\text{peak}}}}$ and [BLa] responses to training, 2 × 5 analysis of variance (ANOVA) were performed, with between-subject factor of “group” (RPE-guided vs PO-guided) and repeated measures on “training session” (1.1 vs 1.3 vs 2.3 vs 3.3 vs 4.3). For %HR_peak_, %PO_peak_, and all perceptual responses, a 2 × 12 ANOVA were performed due to the variables being measured during all training sessions. For all ANOVA the Greenhouse–Geisser epsilon was used unless it was greater than 0.75, in which case the Huynh–Feldt epsilon was used. For assessing the reliability of the %HR_peak_ during training interventions, the coefficient of variation was calculated for each pair of consecutive training sessions (1.1 vs 1.2; 1.2 vs 1.3; 1.3 vs 2.1 etc.) for RPE-guided and PO-guided training groups.

Differences in peak responses to GXT between groups, pre- and post-training were assessed using a two-way mixed measures ANOVA. The within-subject factor was “time” (pre vs post), whilst the between subject-factor was “group” (RPE-guided training vs PO-guided training vs CON).

## Results

### Acute responses to RPE- and PO-guided handcycle training

**Fig. 1:**
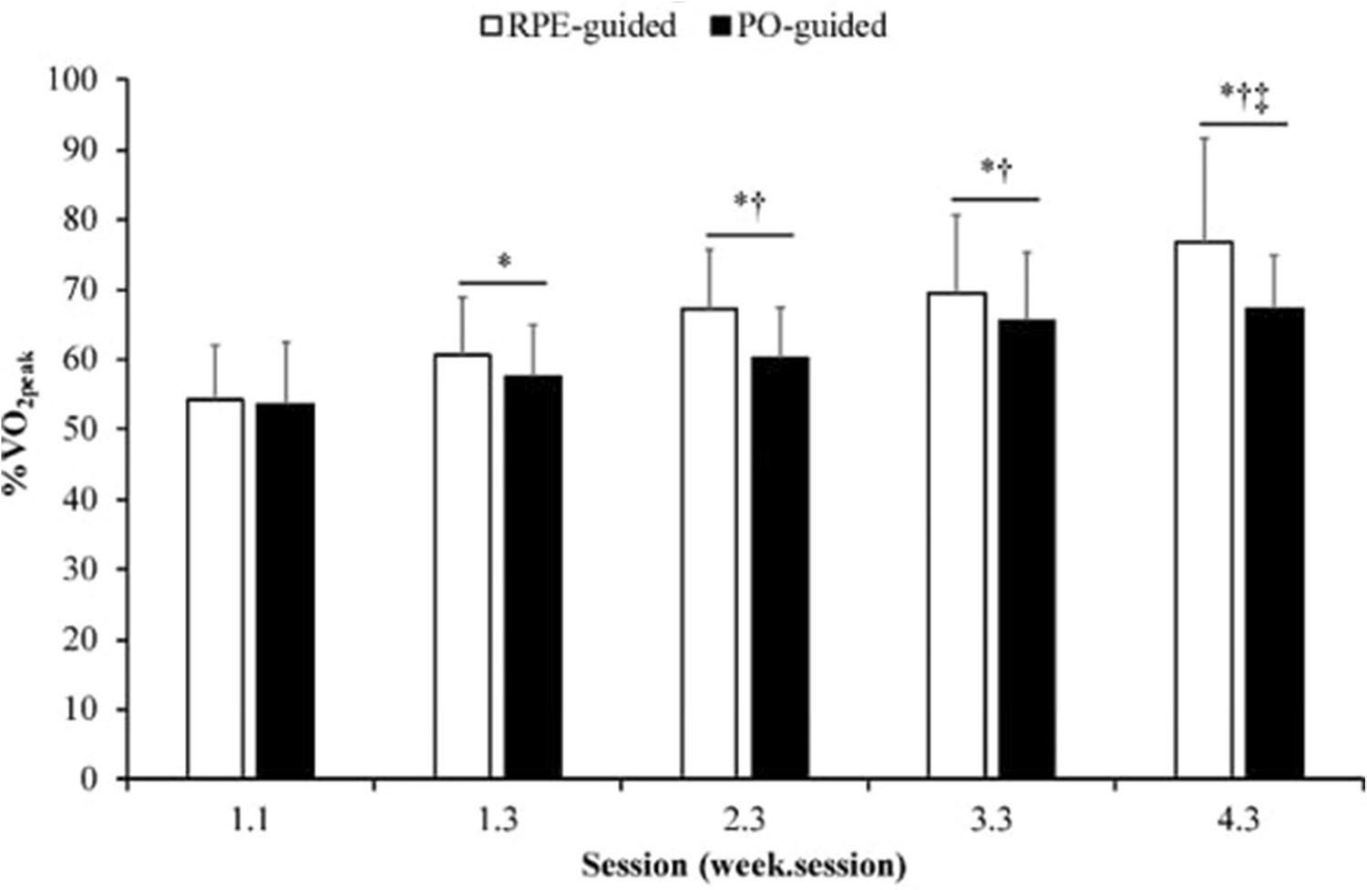
Session-averaged %$\dot{V}O_{{2{\text{peak}}}}$ responses for RPE-guided (white bars) and PO-guided (black bars) training groups as a percentage of pre-training GXT. Data are presented as mean ± SD. *Significantly greater than 1.1; †Significantly greater than 1.3; ‡Sig

The process of matching the %PO_peak_ for the PO-guided (51 ± 6%) group to that produced by the RPE-guided (50 ± 9%) group was successful as there was no difference in mean %PO_peak_ across the 12 training sessions (mean difference, 95% confidence interval: 1, − 7 to 4%; F_(1.00)_ = 0.35, p = 0.56). Similarly, for mean %$\dot{V}O_{{2{\text{peak}}}}$ there was no difference between RPE-guided (66 ± 13%) and PO-guided (61 ± 9%; 5, − 3 to 13%; F_(1.00)_ = 1.62, p = 0.22) training (Fig. 1).

Acute physiological responses across the training intervention are presented in Table 1. There was a main effect of training session on %$\dot{V}O_{{2{\text{peak}}}}$ (F_(2.04)_ = 29.80, p < 0.005), but no significant group-session interaction (F_(2.04)_ = 1.89, p = 0.17). For mean %HR_peak,_ there was no difference between RPE-guided (75 ± 8%) and PO-guided (71 ± 6%; 4, − 1 to 10%; F_(1.00)_ = 2.88, p = 0.11) training across the 12 sessions. There was a main effect of training session on %HR_peak_ (F_(5.32)_ = 11.15, p < 0.005), with no group-session interaction (F_(5.32)_ = 0.82, p = 0.55). Similarly, there was a main effect of training session on [BLa] (F_(2.10)_ = 9.42, p < 0.005), there was no effect of group (F_(1.00)_ = 2.42, p = 0.14), or a group-session interaction (F_(2.10)_ = 1.17, p = 0.32).

**Table 1:** Session-averaged %$\dot{V}O_{{2{\text{peak}}}}$, [Bla]peak, HRpeak, and feeling scale responses for RPE- and PO-guided training groups as a percentage of pre-training GXT

	%$\dot{V}O_{{2{\text{peak}}}}$		%[Bla]_peak_		%HR_peak_		Feeling scale	
	RPE	PO	RPE	PO	RPE	PO	RPE	PO
1.1	54.1 ± 7.8	53.5 ± 8.7	4.3 ± 1.2	4.2 ± 1.0	66.8 ± 7.1	66.4 ± 6.4	1.3 ± 2.0	1.2 ± 1.3
1.2					71.9 ± 7.0	67.7 ± 7.3	1.7 ± 2.2	1.2 ± 1.6
1.3	60.5 ± 8.2*	57.7 ± 7.2*	5.4 ± 1.7*	4.5 ± 0.9	72.1 ± 8.8	69.2 ± 5.4	1.3 ± 1.7	1.1 ± 1.4
2.1					72.9 ± 5.9	67.7 ± 5.4	1.3 ± 1.8	1.2 ± 2.0
2.2					72.8 ± 7.1	69.9 ± 4.2	0.9 ± 1.9	0.8 ± 2.1
2.3	67.0 ± 8.5*†	60.1 ± 7.2*†	6.1 ± 1.9*†	5.0 ± 1.0*	76.0 ± 6.9*	71.3 ± 4.2	1.1 ± 1.7	0.6 ± 1.5
3.1					78.7 ± 8.1*	71.9 ± 3.1	1.2 ± 1.7	0.2 ± 1.7
3.2					76.8 ± 9.1*	72.5 ± 5.6*	1.1 ± 1.8	0.5 ± 2.8
3.3	69.2 ± 11.2*†	65.5 ± 9.5*†	6.2 ± 1.0*†	5.4 ± 1.6*†	78.0 ± 7.8*^+^	74.5 ± 5.5*	1.3 ± 1.8	0.6 ± 1.7
4.1					79.0 ± 7.7*^+^	72.6 ± 4.1*	1.2 ± 1.8	0.7 ± 1.6
4.2					78.0 ± 8.8*^+^	72.5 ± 4.0*	1.5 ± 1.7	0.8 ± 1.4
4.3	76.7 ± 14.9*†‡	67.3 ± 7.4*†‡	6.5 ± 2.1*	5.3 ± 1.2*	78.1 ± 7.8*^+^	73.9 ± 4.5*	1.1 ± 1.8	0.4 ± 1.4

Data are presented as mean ± SD

*PO* PO-guided group, *RPE* RPE-guided group, $\dot{V}O_{2}$ oxygen uptake, [*Bla*] blood lactate, *HR* heart rate

*Significantly greater than 1.1

^+^Significantly greater than 1.2

†Significantly greater than 1.3

‡Significantly greater than 3.3, p < 0.005

Regarding the perceptual responses, there was no difference between RPE-guided and PO-guided training for RPE_O_ (13 ± 0 vs 13 ± 1, p = 0.71), RPE_P_ (14 ± 1 vs 14 ± 1, p = 0.38) or RPE_C_ (12 ± 1 vs 12 ± 1, p = 0.14). The session-averaged FS ratings are presented in Table 1. There was no main effect of group, with FS rating similar between RPE-guided (1.2 ± 1.9) and PO-guided (0.8 ± 1.6; p = 0.48) training groups.

The average coefficient of variation for %HR_peak_ across all comparisons for RPE-guided training (2.8%) was less than PO-guided (3.4%) training, though this was not statistically significant (p = 0.14).

### Chronic responses to handcycle training

Peak responses to the GXT pre and post are presented in Table 2. For absolute $\dot{V}O_{{2{\text{peak}}}}$ there was no main effect of time (F_(1.00)_ = 1.85, p = 0.19), group (F_(2.00)_ = 2.33, p = 0.12), or time by group interaction (F_(2.00)_ = 0.13, p = 0.88). Similarly, for relative $\dot{V}O_{{2{\text{peak}}}}$ there was no main effect of time (F_(1.00)_ = 2.48, p = 0.13) or group (F_(2.00)_ = 2.31, p = 0.12), nor was there a time by group interaction (F_(2.00)_ = 0.21, p = 0.81). For HR_peak_ there was also no main effect of time (F_(1.00)_ = 1.39, p = 0.25), group (F_(2.00)_ = 1.12, p = 0.34) or interaction effect (F_(2.00)_ = 1.95, p = 0.16). For PO_peak_ there was a main effect of time, with PO greater post-training compared to pre-training (F_(1.00)_ = 145.24, p < 0.005). There was no main effect of group (F_(2.00)_ = 1.22, p = 0.31), however the group-time interaction was significant (F_(2.00)_ = 19.71, p < 0.005). The PO_peak_ increased from pre to post in the RPE- and PO-guided training groups, but not in CON.

**Table 2:** Chronic changes in physiological and performance responses to RPE- and PO-guided training interventions

	RPE		PO		Con	
	Pre	Post	Pre	Post	Pre	Post
$\dot{V}O_{{2{\text{peak}}}}$(l/min)	1.9 ± 0.5	2.0 ± 0.6	2.1 ± 0.6	2.1 ± 0.6	1.6 ± 0.4	1.6 ± 0.5
$\dot{V}O_{{2{\text{peak}}}}$(ml/kg/min)	24.0 ± 3.4	25.6 ± 3.9	27.7 ± 5.7	28.3 ± 5.7	23.1 ± 5.1	23.8 ± 5.3
HR_peak_	165.9 ± 18.6	171.1 ± 15.4	163.3 ± 14.2	163.3 ± 12.3	158.8 ± 14.2	158.2 ± 13.5
PO_peak_	130.1 ± 40.2	149.0 ± 43.6*	114.6 ± 35.5	136.4 ± 38.4*	112.3 ± 25.8	116.9 ± 24.1

*Con* non-exercising control group, *PO* PO-guided group, *RPE* RPE-guided group, $\dot{V}O_{2}$ oxygen uptake, *HR* heart rate

*Significantly different from pre, p < 0.005

## Discussion

This is the first study to measure the physiological responses across a long term RPE-guided upper body exercise training intervention in any population. It is also the first to investigate within and between session responses to long-term RPE-guided intervention within the context of existing exercise prescription guidelines. As hypothesised, exercising at a prescription of RPE 13 produced physiological responses aligning with “moderate” to “vigorous” intensity exercise (mean session %$\dot{V}O_{{2{\text{peak}}}}$ = 54–76%; mean session %HR_peak_ = 66–78%), and these responses where not different to a PO-guided exercise intervention in which the prescribed %PO was matched to the mean %PO attained in the RPE training group. However, contrary to the secondary hypothesis, RPE-guided training did not lead to a more positive affective response compared to PO-guided training.

These results support RPE as a primary method of prescribing exercise training intensity over a longer timeframe, which has not previously been explored in such detail (van der Scheer et al. 2016; ACSM 2020). They also demonstrate for the first time that RPE can be used to guide exercise intensity during a chronic handcycling intervention lasting 4-weeks. This is an important finding considering the suitability of moderate to vigorous intensity handcycling for clinicians and practitioners working in a variety of contexts, including exercise prescription for rehabilitation and long-term exercise habits for individuals that have lower limb impairments (e.g., individuals with amputations and spinal cord injuries). This is particularly important with evidence indicating aerobic adaptations occurring after as little as four weeks of handcycling in such populations (Heesterbeek et al. 2005).

The observation that handcycle training at RPE 13 aligned with “moderate” to “vigorous” training intensities builds on previous research, and further supports RPE-guided training. In previously sedentary individuals, it was found that the estimated V̇O_2_ during running sessions at RPE 13 increased over the course of an eight week training intervention from 19.2 ± 1.1 ml/kg/min to 23.4 ± 1.1 ml/kg/min, equivalent to 61 ± 7% of pre-training and 64 ± 7% of post-training $\dot{V}O_{{2{\text{max}}}}$, respectively (Parfitt et al. 2012). In a subsequent study, the average session response over eight weeks of training (3 session per week) prescribed at RPE 13 were estimated to be 74% $\dot{V}O_{{2{\text{max}}}}$ (Parfitt et al. 2015). While different to the presented findings, this may be explained by the way $\dot{V}O_{2}$ was measured. An indirect measure of $\dot{V}O_{2}$ was used in these studies, where $\dot{V}O_{2}$ was estimated based on the speed and gradient of the treadmill during training sessions. Conversely, the current study is the first to use a direct measure of $\dot{V}O_{2}$ to assess the physiological responses over a long term RPE-guided training programme, giving confidence that exercise prescribed at RPE 13 accurately aligned with “moderate” (46–63% $\dot{V}O_{2\max }$) to “vigorous” (64–90% $\dot{V}O_{2\max }$) exercise (ACSM 2020).

In addition to $\dot{V}O_{2}$, the present study is also the first to show the HR response to training at RPE 13 satisfies the classification of “moderate” to “vigorous” intensity in healthy adults performing handcycle exercise. RPE-guided training has been used in recreationally active runners via a weekly 30 min run at the RPE equivalent to ventilatory threshold (Hogg et al. 2018). However, the RPE-guided portion only consisted of a single weekly session of a much wider training programme, with no data showing the intensity of those sessions (Hogg et al. 2018). Thus, the findings do not support the validity of longer-term RPE-guided training within the context of well-established exercise prescription guidelines. Conversely, a month-long exercise intervention in cardiac rehabilitation patients prescribed at RPE 13 was found to produce an average exercise intensity of 64% HR reserve (Ilarraza et al. 2004). This is indicative of “vigorous” intensity exercise, which is higher than the presented findings despite both studies using RPE 13. While uncertain, this may be explained by the different populations explored, where the heart rate response may have been altered in individuals undergoing cardiac rehabilitation (Ilarraza et al. 2004) compared to the healthy adults measured in the current study.

The current findings also serve to support the repeatability of prescribing exercise at an intensity of RPE 13. The average coefficient of variation in the HR response to consecutive training sessions was smaller in the RPE-guided group (2.8%) compared to the PO-guided group (3.4%), though the difference was not statistically significant. The finding of a repeatable HR response to RPE-guided training is vital and could be explained when considering the mechanism behind the generation of the RPE response. A prevailing theory is that RPE during exercise reflects the central motor command arising from pre-motor and motor areas of the brain (Marcora 2009; de Morree et al. 2012). Furthermore, central motor command also contributes to the HR response to exercise via autonomic nervous system activity moderation (Thornton et al. 2002; Nobrega et al. 2014). As such, given that the RPE-guided training group were at a fixed RPE throughout, it would be expected that the HR response would also show limited variability. Though other factors contribute to the HR response to exercise, such as the afferent feedback-induced exercise pressor reflex (Nobrega et al. 2014), results would suggest that using RPE to guide training intensity is a suitable way to ensure a repeatable response to successive, identical sessions. However, it is important to note that the current study was performed in a controlled, laboratory environment and under direct supervision. Although this allows for both rigorous control over the exercise and clear instruction surrounding the use of RPE, it does lack ecological validity. As such, the physiological responses to RPE-guided training could be different in an unsupervised setting. Future studies should investigate these aspects to better understand the intricacies of implementing RPE-guided training beyond laboratory environments.

While the physiological responses to RPE-guided training was valid and reliable, V̇O_2_ and HR increased over the course of the training period (Table 1). Given that prescription was kept at RPE 13, it is unlikely that changes in corollary discharge and afferent feedback explain these observed increases (Abbiss et al. 2015). Instead, this increase may be due to a psychological effect. Exercise intensity tolerance has been defined as the ability to continue exercising when an activity becomes uncomfortable (Ekkekakis et al. 2005). As the participants in the present study were unfamiliar with handcycle exercise, it is possible that as they became accustomed to it, their tolerance to the exercise intensity changed. This result in them “feeling” like they can work at a higher intensity ($\dot{V}O_{2}$, PO) for the same RPE. Although intensity tolerance is often considered to be a stable individual trait not often subject to situational change (Hall et al. 2014), this phenomenon has been observed in interval training settings (Roemmich et al. 2020). As such, this may explain the increase in relative exercise intensity (expressed as a percentage of baseline $\dot{V}O_{{2{\text{peak}}}}$) observed across the training intervention, without an increase in post-intervention $\dot{V}O_{{2{\text{peak}}}}$. It is important to note that as the intervention in the present study was only four weeks, it is unclear whether relative $\dot{V}O_{{2{\text{peak}}}}$ would have continued to increase across a longer duration intervention. Similarly, it is plausible to suggest that over a longer intervention period, larger and statistically significant increases in $\dot{V}O_{{2{\text{peak}}}}$ may be observed, which would partially explain these findings.

Interestingly, there was no difference in affect between RPE- and PO-guided training. This contrasts the hypothesis that RPE-guided training would lead to a more positive affective response. Previous research has suggested that exercising at self-selected intensities leads to a more positive affective response than prescribed exercise intensities, such as that undertaken by the PO-guided exercise group in the present study, who did not have the autonomy to choose their own training loads (Rose and Parfitt 2007; Hamlyn-Williams et al. 2014). However, it can be argued that for the RPE-guided group the intensity was not truly self-selected, as the PO chosen by the participant had to correspond with a specific, prescribed, RPE. Therefore, the similar affect between groups is likely a result of the similar exercise intensity of the training interventions. It is also important to highlight that while FS rating during exercise was positive for both groups, though there was a large degree of interindividual variation. This variation could be the result of participants having an individual “intensity preference,” in which they may have a known (or unknown) preference for a particular exercise intensity. Intensity preference has been shown to influence FS ratings during exercise, which could contribute to this finding (Ekkekakis et al. 2005). Taking this into consideration, the comparison of affect between training groups is limited due to the inability to control for intensity preference. Future studies should incorporate measures of exercise tolerance and intensity preference to provide a greater understanding of how they impact affective responses to RPE-guided training.

Previous investigations into long-term RPE-guided training have been limited to lower-body exercise modalities. This is the first study to investigate the physiological response to a chronic RPE-guided handcycle programme. For able-bodied participants, upper body endurance exercise provides an alternative to common lower-body exercise modes. For those with mobility issues or disability of the lower limb (e.g., amputees or spinal cord injuries), upper body endurance exercise offers an ecologically valid method of improving cardiovascular endurance and health. In participants with spinal cord injury, RPE has been used to guide exercise intensity during training interventions (Pelletier et al. 2015; Kim et al. 2015; Valent et al. 2010; Nooijen et al. 2015; Totosy de Zepetnek et al. 2015; Bakkum et al. 2015; van der Scheer et al. 2016). However, the produced physiological responses to that training have not been presented. As such, the present study lays the groundwork for further research regarding RPE-guided training in populations where upper body endurance modalities are the primary form of exercise.

There are some limitations that should be considered when interpreting these findings. Firstly, while the RPE- and PO-guided groups were matched for gender, CON was not, which resulted in CON having twice as many females as other groups. There is the potential that this could have led to differences in habitual exercise habits between the groups, although as there was no change in chronic physiological parameters, any potential differences were unlikely meaningful. Secondly, $\dot{V}O_{{2{\text{peak}}}}$ was used as a measure of maximal fitness in this study, whereby all GXT’s ceased at volitional exhaustion, and maximal oxygen uptake was not validated. Although GXT’s generally yield a highly reproducible measures of maximal aerobic fitness irrespective of exercise test protocol (Poole and Jones 2017), maximal aerobic fitness may have been underreported in the present study. Lastly, the population studied was restricted to healthy able-bodied adults. As such, these findings may not translate directly to those populations likely to benefit most from handcycling exercise (e.g., individuals with amputations and spinal cord injuries), who may demonstrate different physiological responses to handcycling exercise than able-bodies individuals.

## Conclusion

This is the first study to document the physiological responses to a novel RPE-guided handcycle training programme in able-bodied adults. While $\dot{V}O_{{2{\text{peak}}}}$ did not increase significantly across the 4-week intervention, the results suggest that training at an intensity of RPE 13 is a repeatable and produces a physiological response indicative of moderate to vigorous intensity. Furthermore, although RPE-guided training did not lead to a more positive affective response than PO-guided training, affect was positive. This study provides supports the use of RPE to guide exercise intensity during upper body exercise training programmes.

## Data Availability

Data available upon reasonable request from the first author, Michael Hutchinson (mike.hutchinson@paralympics.org.uk).
