# Correction to: Oxygen uptake and heart rate responses to 4 weeks of RPE‑guided handcycle training

**DOI:** 10.1007/s00421-023-05229-w

**Published:** 2023-06-05

**Authors:** Michael J. Hutchinson, Thomas A. W. Paulson, Christof A. Leicht, Hunter Bennett, Roger Eston, Victoria L. Goosey-Tolfrey

**Affiliations:** 1 British Paralympic Association, London, UK; 2 Sport Exercise and Health Sciences, The Peter Harrison Centre for Disability Sport, Loughborough University, Loughborough, UK; 3 UK Athletics, Birmingham, UK; 4 Allied Health and Human Performance, University of South Australia, Adelaide, Australia; 5 Alliance for Research in Exercise, Nutrition and Activity (ARENA), University of South Australia, Adelaide, Australia

**Correction to: European Journal of Applied Physiology** 10.1007/s00421-023-05210-7

The original version of this article unfortunately contained a mistake. Affiliation details for Author Thomas A. W. Paulson were incorrectly given

^1^British Paralympic Association, London, UK

^3^UK athletics, Birmingham, UK

but should have been

^2^Sport Exercise and Health Sciences, The Peter Harrison Centre for Disability Sport, Loughborough University, Loughborough, UK

^3^UK athletics, Birmingham, UK

